# Odd electron diffraction patterns in silicon nanowires and silicon thin films explained by microtwins and nanotwins

**DOI:** 10.1107/S0021889808042131

**Published:** 2009-01-24

**Authors:** Cyril Cayron, Martien Den Hertog, Laurence Latu-Romain, Céline Mouchet, Christopher Secouard, Jean-Luc Rouviere, Emmanuelle Rouviere, Jean-Pierre Simonato

**Affiliations:** a CEA DRT, LITEN, Minatec, 38054 Grenoble, France; b CEA, DSM, INAC, Minatec, 38054 Grenoble, France

**Keywords:** silicon nanowires, silicon thin films, artifacts, twinning

## Abstract

Odd electron diffraction patterns (EDPs) have been obtained by transmission electron microscopy (TEM) on silicon nanowires grown *via* the vapour–liquid–solid method and on silicon thin films deposited by electron beam evaporation. Many explanations have been given in the past, without consensus among the scientific community: size artifacts, twinning artifacts or, more widely accepted, the existence of new hexagonal Si phases. In order to resolve this issue, the microstructures of Si nanowires and Si thin films have been characterized by TEM, high-resolution transmission electron microscopy (HRTEM) and high-resolution scanning transmission electron microscopy. Despite the differences in the geometries and elaboration processes, the EDPs of the materials show great similarities. The different hypotheses reported in the literature have been investigated. It was found that the positions of the diffraction spots in the EDPs could be reproduced by simulating a hexagonal structure with *c*/*a* = 12(2/3)^1/2^, but the intensities in many EDPs remained unexplained. Finally, it was established that all the experimental data, *i.e.* EDPs and HRTEM images, agree with a classical cubic silicon structure containing two microstructural defects: (i) overlapping Σ3 microtwins which induce extra spots by double diffraction, and (ii) nanotwins which induce extra spots as a result of streaking effects. It is concluded that there is no hexagonal phase in the Si nanowires and the Si thin films presented in this work.

## Abstract 2

Anomalous extra spots visible in electron diffraction patterns of silicon nanowires and silicon thin films are explained by the presence of micro- and nanotwins.

## 1. Introduction

Si nanowires (NWs) are low-dimensional objects with promising potential in the new, emerging semiconductor industries. Si NWs could constitute the interconnects and functional components of the future microelectronic industry (Cui & Lieber, 2001 ▶). They can also be integrated into photovoltaic solar cells (Tian *et al.*, 2007 ▶) or thermoelectric devices (Hochbaum *et al.*, 2008 ▶; Boukai *et al.*, 2008 ▶) to enhance power conversion efficiencies and have a role to play in the new energy industries. Polycrystalline Si thin films have been developed for more than 15 years for logic circuit and videographic applications (Brotherton *et al.*, 1991 ▶), and for first- and second-generation photovoltaic solar cells (Aberle, 2006 ▶).

Both Si NWs and Si thin films are developed at CEA–Grenoble for new energy applications. This study began by a simple process control of Si NWs by transmission electron microscopy (TEM). We were unable to index the electron diffraction patterns (EDPs) of many Si NWs and were therefore unable to determine their growth directions. We decided that it was necessary to improve our understanding of these odd EDPs. There are very few studies in the literature on odd EDPs in Si NWs; however, peculiar EDPs are often reported in Si thin films. It was unclear whether the odd EDPs in Si thin films were the same as those of Si NWs. Odd EDPs were first reported by Lassen (1934 ▶) for face centred cubic (fcc) metals deposited by vacuum evaporation, but their origins have not been fully clarified and many hypotheses exist to explain them. Each is considered in detail below.

### 1.1. Size effect

The extra spots in the odd EDPs of thin fcc films have been interpreted by Cherns (1974 ▶) as an effect of the finite sequence of the *ABC* layers of the (111) planes. In very thin objects, the sequence is incomplete, *i.e.* is not a multiple of three (111) planes, and some hexagonal compact packings (hcp) remain at the surfaces of the object. They correspond to a 2H structure and explain the anomalous spots in the EDPs. Cherns’ interpretation was used to explain odd diffraction in silicon thin films (Gibson *et al.*, 1989 ▶) and in silicon NWs (Bell *et al.*, 2004 ▶) (Si can be considered as the sum of two translated fcc structures). Based on the same idea, Korgel *et al.* (2006 ▶) simulated odd EDPs in Si NWs of diameters less than 10 nm. However, Cherns’ interpretation cannot explain the anomalous spots observed in the EDPs of silicon films or nanowires of thickness or diameter greater than 100 nm.

### 1.2. Twinning effect

**Figure 1:**
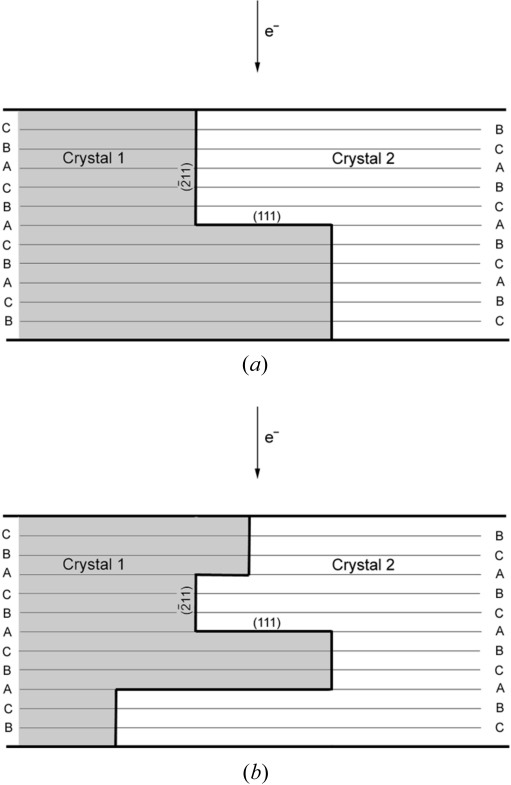
Double positioning grain boundary in fcc thin film: overlapping of the two twinned crystals along the electron direction 〈111〉. (*a*) Schematic representation (from Dickson & Pashley, 1962 ▶). Pashley & Stowell (1963 ▶) used this representation and other arguments based on complex double diffraction between non-excited spots to explain the extra spots at 1/3{422} in the 〈111〉 EDPs. (*b*) We propose another schematic representation of the double positioning grain boundary. Nano-interpenetrating steps at the interface act as planar defects on the {111} planes and induce streaks along the reciprocal vector **g**_(111)_. The streaks intersect the Ewald sphere and create the extra spots at 1/3{422} (details in §§3.1 and 5.2).

Odd EDPs in gold thin films were explained by Dickson & Pashley (1962 ▶) and by Pashley & Stowell (1963 ▶) as double diffraction between superposed twinned crystals. Such superposition, called ‘double positioning’, was observed by dark-field imaging and found to occur at the grain boundaries of the film. A schematic representation is given in Fig. 1 ▶(*a*). However, some anomalous spots, such as the extra spots situated at 1/3{422}, could be explained only by introducing an unusual notion of double diffraction between non-excited spots. Buffat *et al.* (1991 ▶) reported odd power spectra of HRTEM (high-resolution transmission electron microscopy) images of gold nanoparticles, and these authors could prove, by convincing image simulations, that the anomalous spots were due to a superposition of twins and did not result from an hcp phase. Carim *et al.* (2001 ▶) found one odd power spectrum in an Si NW oriented in the [111] direction, and they interpreted it as the consequence of a twin lying along the growth direction of the nanowires. Kohno *et al.* (2003 ▶) established, by convincing simulations, that some extra spots in silicon nanoparticles are due to the diffraction of microtwins, and that others, such as the 1/3{422} extra spots, are induced by streaking effects probably produced by stacking faults (and not by complex double diffraction as previously imagined by Pashley & Stowell, 1963 ▶).

Pashley & Stowell (1963 ▶), Buffat *et al.* (1991 ▶) and, more recently, Kohno *et al.* (2003 ▶) warned the scientific community not to interpret the odd EDPs by assuming the existence of hexagonal silicon phases. However, despite their warning, as will be seen in the next section, a very high number of papers still mention new phases in Si NWs and Si thin films.

### 1.3. Hexagonal silicon structures

More than 12 forms, including the wurtzite 2H structure, have been reported since 1963 for silicon under high pressure (Wentorf & Kasper, 1963 ▶; Besson *et al.*, 1987 ▶). These studies led researchers to think that such phases could also exist in metastable silicon elaborated by physical or chemical vapour deposition processes.

In silicon thin films, the presence of hexagonal phases has been widely accepted and reported for more than 20 years. Hendriks *et al.* (1984 ▶) studied by X-ray analysis Si thin films elaborated by low-pressure chemical vapour deposition (LPCVD). From new peaks in the angular profiles close to the classical *d*_(111)_, these authors proposed the existence of Si polytypes resulting from a high density of stacking faults or microtwins. Dahmen *et al.* (1989 ▶) observed by TEM the existence of 2H silicon at the intersection of two deformation microtwins in a silicon matrix. A disclination model was later proposed by Müller & Pirouz (1997 ▶) to explain the formation of this 2H structure. Cerva (1991 ▶) confirmed by HRTEM observations the existence of the 2H Si structure in LPCVD Si thin films and also observed other 4H and 9R polytypes. The presence of these polytypes was explained by a stress-induced martensitic transition at an intersection of crossing twins, according to the model proposed by Dahmen *et al.* (1989 ▶). Since then, other studies have reported the existence of the 2H structure in LPCVD Si thin films by TEM observations (Nakhodkin *et al.*, 2000 ▶) or by Raman experiments (Bandet *et al.*, 2000 ▶).

In these studies, the 2H polytype appears as a sequence of stacking faults locally ordered on a scale of a few nanometres. Higher ordering distances can be obtained by artificially controlling the stacking fault sequence by using multi-step molecular epitaxy on Si(111) surfaces and boron as surfactant twinning inducer (Fissel *et al.*, 2006 ▶). Many studies also report the existence of hexagonal forms of silicon in CVD (chemical vapour deposition) thin films recrystallized by laser excitation. Marfaing & Marine (1989 ▶) indexed four EDPs and proposed a hexagonal structure with *a* = 0.39 and *c* = 0.94 nm. These authors did not notice that the *c*/*a* ratio of 2.43 is very close to 3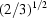. This result is strange because the sequence length usually corresponds to the cubic diamond 3C phase. Another hexagonal structure with a *c*/*a* ratio close to 2.68 was obtained later by Kim & Lee (1996 ▶). More recently, Zhang *et al.* (1999 ▶) observed odd EDPs in Si thin films deposited by laser ablation and proposed a simple 2H structure with, however, a large discrepancy between the experimental and calculated diffraction spot positions (> 5%). Very similar odd EDPs were also observed in germanium thin films deposited by CVD and recrystallized by electron beam (Parsons & Hoelke, 1984 ▶) or by laser excitation (Cesari *et al.*, 1985 ▶) techniques, or deposited by laser ablation (Zhang *et al.*, 2000 ▶). In Si NWs, some papers also assume the existence of hexagonal phases to explain the odd EDPs. Fontcuberta i Morral *et al.* (2007 ▶) indexed two unusual HRTEM images in Si NWs with a 2H Si structure (with an error close to 5%). A deeper TEM study had already been performed on Si whiskers by Miyamoto & Hirata (1978 ▶) – at that time, nanowires were called ‘whiskers’ – and these authors indexed four EDPs with an error far lower than 5% with a 6H Si structure; they also reported other hexagonal and rhombohedral polytypes of Si (27R, 51R and 141R).

**Figure 2:**
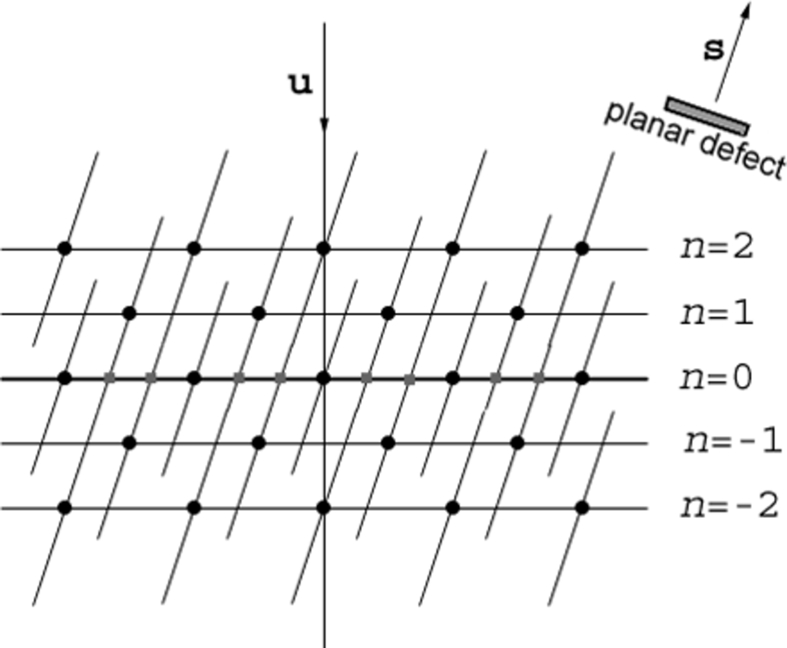
Electron diffraction conditions of a crystal containing planar defects. The electron beam follows the crystalline direction **u**. The diffraction spots are the vectors **g** of the reciprocal lattice that intersect the Ewald sphere; they are given by the condition **u**  **g** = 0 (zero-order Laue zone). The vectors **g** of higher-order Laue zones **u**  **g** = *n* are not in diffraction condition but, in the presence of planar defects, they can be degenerated along the direction **s** normal to the defect plane and the vectors **g** + *k* **s** with *k* ∈ [−1, 1] intersect the Ewald sphere when (**g** + *k* **s**) = 0.

What is striking in this short review is that all the odd EDPs reported in the literature seem to be very similar, whatever the nature of the silicon and the elaboration process. For example, the power spectrum Fig. 2f given by Fontcuberta i Morral *et al.* (2007 ▶) in Si NWs looks the same as the EDP of (i) Fig. 2 of Dickson & Pashley (1962 ▶) in a gold thin film, (ii) Fig. 4b of Buffat *et al.* (1991 ▶) in a gold nanoparticle, (iii) Fig. 4b of Kohno *et al.* (2003 ▶) in an Si nanoparticle, (iv) Fig. 3b of Miyamoto & Hirata (1978 ▶) in an Si whisker, (v) Fig. 1b of Marfaing & Marine (1989 ▶) in an LPCVD Si thin film, and (vi) Fig. 1c of Zhang *et al.* (2000 ▶) in laser ablation Si thin film. This pattern was explained by the presence of hexagonal phases by Marfaing & Marine (1989 ▶), Zhang *et al.* (2000 ▶) and Fontcuberta i Morral *et al.* (2007 ▶), and by a superposition of two diffraction patterns produced by two twinned 3C crystals in [123]_crystal 1_ = [321]_crystal 2_ zone axes by Dickson & Pashley (1962 ▶), Buffat *et al.* (1991 ▶) and Kohno *et al.* (2003 ▶). Another example is the [111]_3C_ zone axis EDP with 1/3{422} extra spots given in Fig. 1i of Kohno *et al.* (2003 ▶), Fig. 10 of Pashley & Stowell (1963 ▶), Fig. 3b of Carim *et al.* (2001 ▶) and Fig. 1d of Zhang *et al.* (1999 ▶). This pattern was explained by hexagonal phases by Zhang *et al.* (1999 ▶), complex double diffraction by Pashley & Stowell (1963 ▶), and a streaking effect by Kohno *et al.* (2003 ▶).

In order to provide a good explanation for the odd EDPs in Si NWs and Si thin films, we have fully characterized these materials by TEM and HRTEM and simulated as far as possible the different solutions proposed in the literature. We will show that the odd EDPs result from both microtwinning and nanotwinning. This study will therefore confirm and also complete and correct some details of the Pashley & Stowell (1963 ▶) and Kohno *et al.* (2003 ▶) studies.

## 2. Experiments

The Si NWs of this study were elaborated by CVD using the vapour–liquid–solid (VLS) method (Wagner & Ellis, 1964 ▶) with gold as catalyst and SiH_4_ or SiH_2_Cl_2_ diluted in H_2_ as gaseous precursors at temperatures between 773 and 1023 K under a pressure of 10 Torr (1 Torr = 133.322 Pa). Details of the process are given elsewhere (Latu-Romain *et al.*, 2008 ▶). The Si thin films (2 µm thick) were deposited by electron beam evaporation onto silicon nitride coated glass substrates. They were recrystallized in a tubular quartz furnace under an argon atmosphere at 873 K.

For the preparation of the TEM specimen, the Si NWs were dispersed in ethanol with ultrasonic apparatus and deposited onto a Cu grid covered by a thin amorphous carbon layer. A cross section of one Si NW was also prepared by focus ion beam (FIB) milling using FEI Strata400 equipment. The Si thin films were prepared by classical cross section, *i.e.* mechanical polishing and ion milling. The microstructural observations were performed by conventional TEM with a JEOL 2000FX (200 kV), by HRTEM with a JEOL 4000EX (400 kV) and by high-resolution scanning transmission electron microscopy (HRSTEM) with an FEI Titan (300 kV) equipped with a condenser Cs corrector and a high-angle annular dark-field (HAADF) detector. The EDPs were obtained on the 2000FX TEM by using double-tilt and rotation–tilt sample holders. The power spectra (*i.e.* the square of the fast Fourier transform magnitude of HRTEM images) were calculated using a digital micrograph.

## 3. Simulations of the diffraction patterns

A simple computer program has been written to simulate the EDPs for any crystallographic structure. There are many other and more powerful computer programs, such as *JEMS* (Stadelmann, 1987 ▶), but our program has the advantage of being integrated in a larger piece of software, called *GenOVa* (Cayron, 2007*b* ▶), dedicated to the calculation of variants. The simulations are purely kinematical. The dynamical effects are not taken into consideration; however, optionally, the extra spots that could come from a double diffraction can be calculated by linear combinations. Other specific points are detailed in the following sections.

### 3.1. Hexagonal silicon polytypes and their relationship with the cubic phase

*GenOVa* calculates the diffraction patterns of crystals linked by an orientation relationship (OR), and it superposes these diffraction patterns. The OR between an ideal polytype of type *n*H (for *n* even) or *n*R (for *n* odd) and the usual cubic phase 3C is 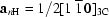 and 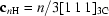. The ideal polytype *n*H (or *n*R) has lattice parameters 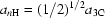 and 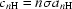, where 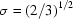. For example, *c*_2H_/*a*_2H_ = 2σ in the wurtzite structure. In the following, the direct basis of the cubic crystal, denoted DIR_3C_, is normalized. The transformation matrix from DIR_3C_ to the direct basis DIR_*n*H_ of the *n*H polytype crystal is given by 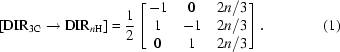
The metric tensor of the polytype *n*H, *i.e.* the transformation matrix from the reciprocal basis to the direct basis of the polytype, is expressed by the matrix 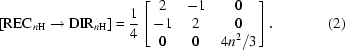
Since [REC_3C_ → DIR_3C_] is the identity matrix (because the reference frame DIR_3C_ is normalized), the transformation matrix from the reciprocal basis REC_3C_ of the cubic crystal to the reciprocal basis REC_*n*H_ of the *n*H polytype crystal is [REC_3C_ → REC_*n*H_] = [REC_3C_ → DIR_3C_][DIR_3C_ → DIR_*n*H_] × [DIR_*n*H_ → REC_*n*H_] = [DIR_3C_ → DIR_*n*H_][REC_*n*H_ → DIR_*n*H_]^−1^ and, consequently, 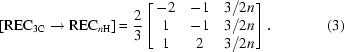
The matrices presented in this section are automatically calculated by *GenOVa*. They are used in the simulations of the EDPs of the polytypes.

### 3.2. Streaking effects

The computer program also calculates the extra spots created by diffraction relaxation effects. Indeed, stacking faults or other planar defects in a crystal degenerate the nodes of the reciprocal lattice along a vector **s** normal to the defect plane. If the defect is thin, the streaks are elongated and can intersect the Ewald sphere to produce ‘extra’ diffraction spots as shown in Fig. 2 ▶. The algorithm used to calculate the positions of these extra spots is now detailed. The electron beam is parallel (no convergence), and the Ewald sphere is reduced to a plane owing to the small wavelength of electrons in comparison with the interplanar distances. Consequently, only diffraction in the zero-order Laue zone is considered in the simulations. A (111) platelet defect of thickness *n* *d*_(111)_ induces a degeneracy vector 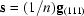. For a given zone axis **u**, the number *N* of Laue zones that intersect the Ewald sphere as a result of the streaking effect is given by the product of the norms 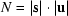. The vectors **g** that belong to these high-order Laue zones (HOLZs) are given by vectors with integer coordinates in accordance with the equation 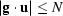. Any vector **g** of this list is degenerated and forms a segment given by **g **± *k* **s** where *k* ∈ [0, 1]. The segment intersects the Ewald sphere for a real number *k* ∈ [0, 1] such that **u**  (**g ** ±  *k* **s**) = 0. The numbers *k* are easily calculated and the vectors **g ** ±  *k* **s** constitute the extra spots produced by the streaking effect.

### 3.3. Twins in a cubic silicon matrix

In this study, we were also particularly interested in twins. Twins in fcc and diamond structures are (111) mirror symmetries. The orientation relationship between twinned crystals is often denoted Σ3 because the two lattices have one-third of their nodes in common (Bollman, 1970 ▶, 1982 ▶). Twins are frequently observed in low stacking fault energy materials such as Cu (20–50 mJ m^−2^), but they are also frequent in silicon despite its higher stacking fault energy (50–100 mJ m^−2^). Twinning mechanisms in silicon are different from those of copper. In copper, twins are formed during annealing and recrystallization treatments, whereas in silicon thin films, twins are probably generated by the shear stresses induced by the amorphous–crystalline transformation. In Si NWs, twins are not yet clearly understood, but could result from the stresses induced by the fast liquid–solid transformation at the droplet/nanowire interface, or from the possible presence of impurities in the liquid that modify the stacking fault energy of silicon and segregate at the twin boundaries, such as gold atoms (Allen *et al.*, 2008 ▶). The boundary between two twinned crystals is not always the (111) mirror plane. If necessary, one must specify the interfacial plane in the notation; for example, a Σ3{112} is a Σ3 twin with an interface plane of type {112} [such interfaces have been observed, for example, in cast silicon by Kuchiwaki *et al.* (2005 ▶)]. A silicon crystal has four twinned variants as a result of its four {111} equivalent planes that act as mirror symmetries. Algebraically, the orientations of the four variants are given by sets of equivalent matrices **g**_*i*_ **T** with *i* ∈ {0, 1,…, 3}, where **T** is the matrix representing the (111) mirror symmetry and **g**_*i*_ are symmetry matrices arbitrarily chosen in the four cosets of the quotient set **G**/**H**, where **G** is the point group of silicon (*m*3*m*) and **H** is the intersection group. In general, the identity matrix is chosen for **g**_0_. Theoretical details are given elsewhere (Cayron, 2007*a* ▶). The four matrices chosen in this study are 
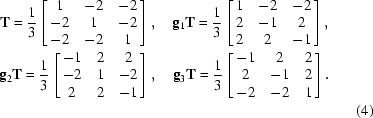
They are transformation matrices from a basis of a reference crystal to the bases of its four twinned variants. We use their inverses to calculate the direction of the electron beam in the bases of the twinned crystals. For example, the [011] zone axis in a silicon crystal becomes [411], [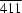], [0
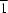
1] and [01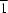] zone axes for the four twins. Since, in this study, we consider that twinning only occurs in the diamond Si phase (cubic), the metric tensor is reduced to the identity matrix and the four matrices [equation (4)] can also be used to calculate the positions of the diffraction spots of the twin variants in the reference frame of their parent crystal (this parent crystal is also, in general, the matrix in which the twin variants are embedded).

In the following, we will use the term ‘microtwin’ to refer to a twin inside a parent matrix with size ranging from 30 nm to micrometres. The microtwins are large enough to diffract as normal independent crystals but can also produce extra spots by double diffraction with their parent crystal (§3.3). We will use the term ‘nanotwins’ to refer to very thin twins lying on the {111} planes, with thickness ranging from one to ten times *d*_(111)_. Such nanotwins are nearly two-dimensional objects that generate the diffraction spots of the parent crystal by a streaking effect (§3.2). The term ‘nanotwins’ includes the stacking faults on the {111} planes. All the simulations of the EDPs (hexagonal phases, micro- and nanotwins) were performed with our software *GenOVa* (Cayron, 2007*b* ▶).

## 4. Experimental results

### 4.1. Silicon nanowires

**Figure 3:**
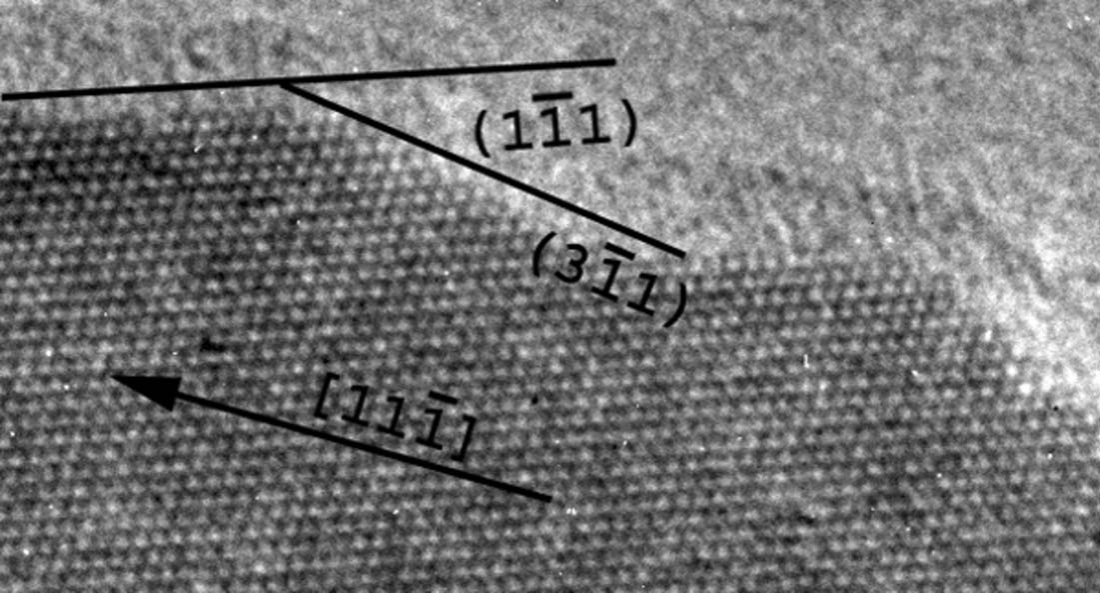
HRTEM image of the {111} and {113} edges of an Si NW that grows in the [11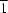] direction (in the plane of the page).

**Figure 4:**
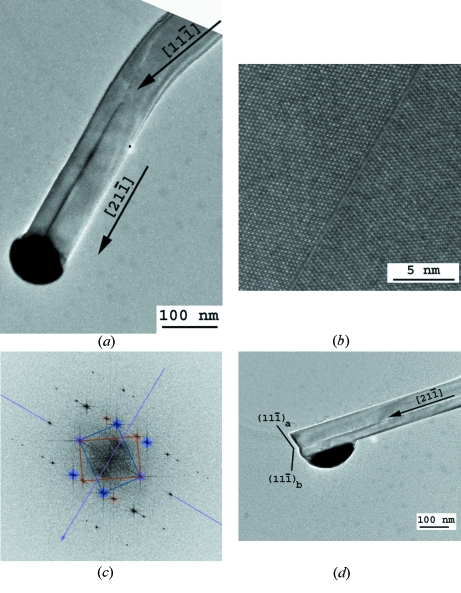
Bicrystalline Si NWs. (*a*) TEM image of a bent Si NW that changes its growth direction from 〈111〉 to 〈211〉 by twinning. The electron beam is along the [011] zone axis. (*b*) HRTEM image acquired in the middle of the bicrystal and (*c*) the corresponding power spectrum of the fast Fourier transform. (*d*) Another Si NW: after twinning, the position of the Au–Si droplet became unstable because of the angle between the new growth direction 〈112〉 and the {111} planes of the two crystals (denoted *a* and *b*), and the droplet topples to the side of the thinner crystal (here *b*).

**Figure 5:**
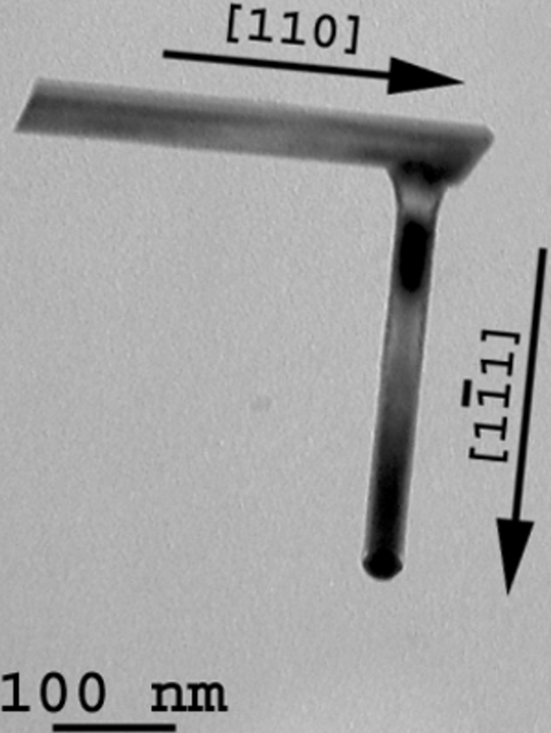
Formation of a 90° elbow in an Si NW. Such a shape can be explained by a toppling effect. First, the nanowire grows in the 〈110〉 direction, but, because of the angle between the {111} tip surface and the growth direction, the droplet topples at the edge of the nanowire. The nanowire continues to grow in epitaxy with the 〈110〉 nanowire but in a 〈111〉 direction.

**Figure 6:**
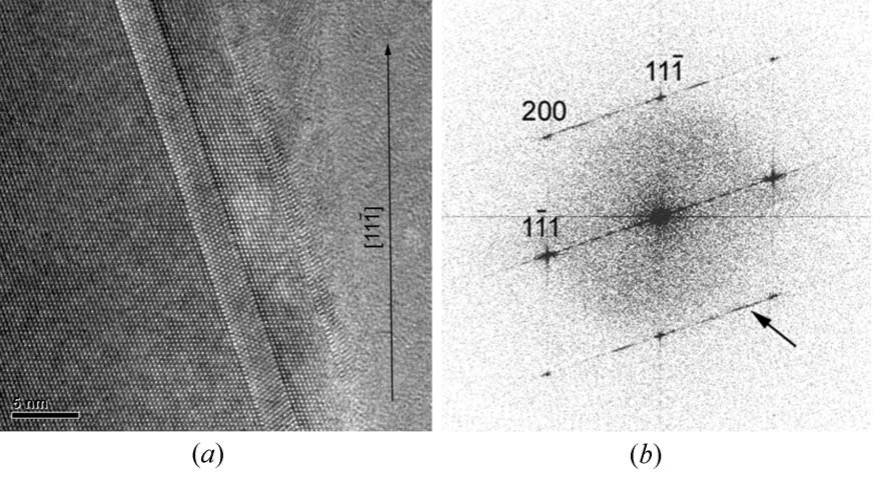
Nanotwin in an Si NW in the [011] zone axis. (*a*) HRTEM image and (*b*) power spectrum of the image. The growth direction is [11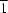] and the twin mirror plane is (1
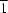
1). The nanotwin has a thickness of nine (111) planes, which produces streaks in the power spectrum; one is marked by an arrow.

Most of the Si NWs are straight single crystals with diameters that vary from 20 to 500 nm. From the EDPs acquired on untilted nanowires and easily indexed by the diamond structure, we could establish that the growth direction is generally 〈111〉, quite often 〈112〉 and occasionally 〈110〉. We could not observe any relationship between the growth directions and the diameters of the nanowires. The external surfaces of the Si NWs are constituted of {111} and {311} facets (Fig. 3 ▶), which correspond to the planes of lowest energy for the diamond Si structure. More than 40% of the nanowires are twinned along their growth direction (Fig. 4 ▶). These NWs are bent by twinning, they become bicrystalline and their growth direction changes from 〈111〉 to 〈211〉 as shown in Fig. 4 ▶(*b*). The new 〈211〉 growth direction is common to both crystals that constitute the bicrystal. The bend angle is the angle between a 〈111〉 and a 〈112〉 direction and is equal to 19.5°. When this kind of twin occurs, the droplet becomes unstable because the {111} tip plane on which the droplet is lying degenerates into two different {111} planes of the two twinned crystals. Sometimes, the gold droplet topples to the side of the thinner crystal and the Si NW stops growing as illustrated in Fig. 4 ▶(*d*). Such a toppling effect can also be observed in untwinned Si NWs that grow along directions different from 〈111〉 and may create elbow shapes as shown in Fig. 5 ▶. Twinning can also lead to multi-branched Si NWs with different 〈111〉 growth directions (not shown). Some structural defects were found in the Si NWs, such as stacking faults and nanotwins, as shown in Fig. 6 ▶.

**Figure 7:**
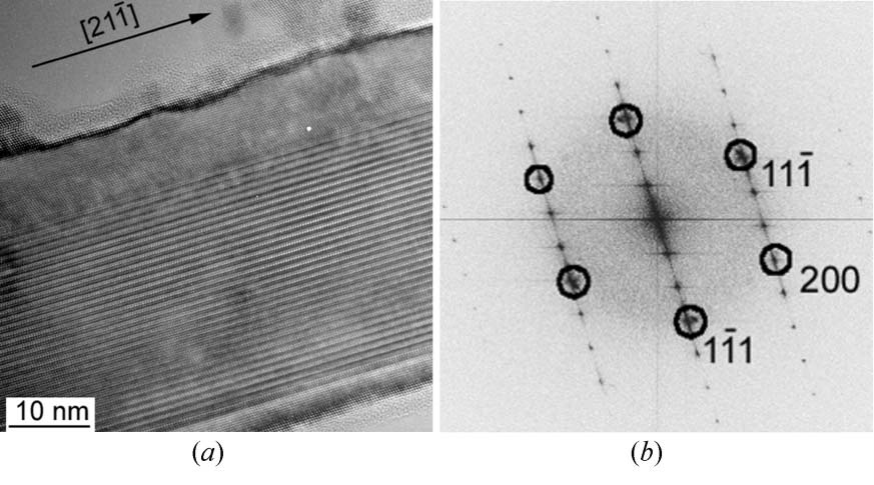
Odd feature in an Si NW in the [011] zone axis. (*a*) HRTEM and (*b*) power spectrum. The superstructure-like pattern follows the [21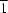] growth direction of the NW. A periodicity of 3*d*_(111)_ is clearly visible in both the image and the power spectrum. It is shown in §5.2 that such a pattern is, in fact, a Moiré effect produced by overlapping twins.

**Figure 8:**
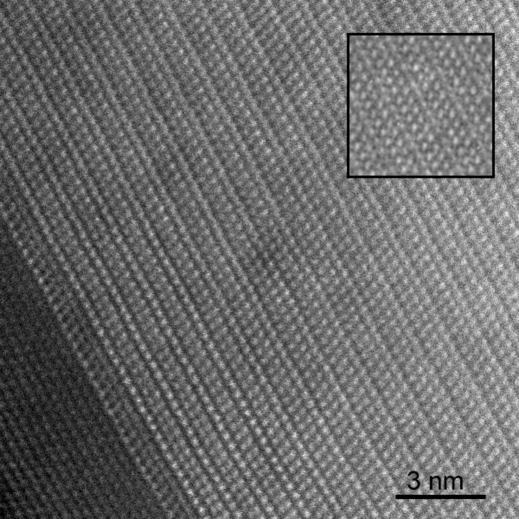
HRSTEM image of a superstructure-like pattern in an Si NW growing along the [21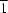] direction. The superlattice seems to have a periodicity of 3*d*_(111)_, and atomic columns inside the lattice are clearly resolved. In the top right corner is a ‘simulation’ of this superstructure-like pattern that consists of a superposition of the twinned parts of Fig. 4 ▶(*b*), as explained in §5.2.

Many Si NWs exhibit particular EDPs whatever their size. Details of these EDPs are given in §4.3. In order to understand their structure better, HRTEM observations were carried out. An Si NW composed of a mixed structure along its 〈112〉 growth direction is presented in Fig. 7 ▶; the two sides have a normal Si structure, but the centre looks like a superstructure with a periodicity of 3*d*_(111)_. Similar periodic patterns with clear atomic resolution were also obtained by HRSTEM (Fig. 8 ▶). Such images could lead us to believe that a silicon superstructure exists in the Si NWs, but we will see in the following sections that this is not the case.

### 4.2. Silicon thin films

**Figure 9:**
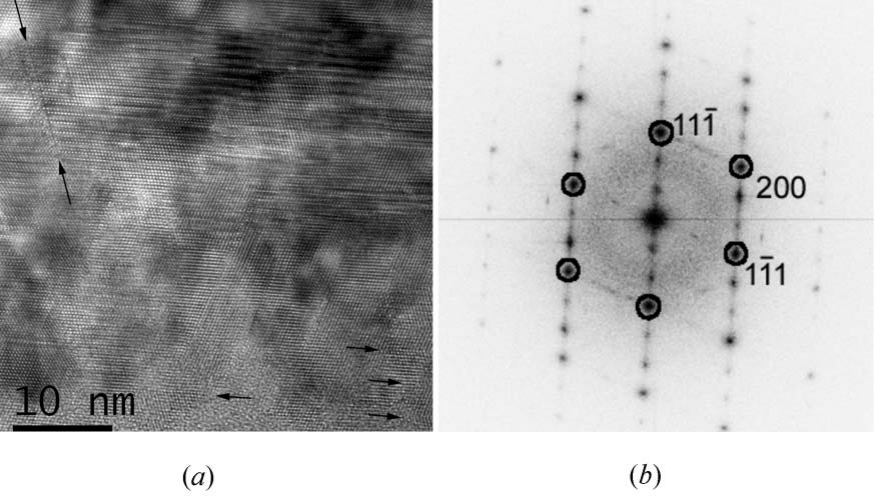
Cross section of an Si thin film in the [011] zone axis. (*a*) HRTEM image and (*b*) power spectrum. The odd feature with a periodicity of 3*d*_(111)_ similar to Fig. 7 ▶ is visible in the thicker part of the sample (centre of the image). Its left side, marked by the two inclined arrows, corresponds to a vertical (13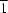) plane. The atomic columns cannot be clearly distinguished. The feature is actually a Moiré effect produced by the superposition of twinned crystals. In the thinner parts of the sample, alternating (but not superposing) twins are indicated by the horizontal arrows.

**Figure 10:**
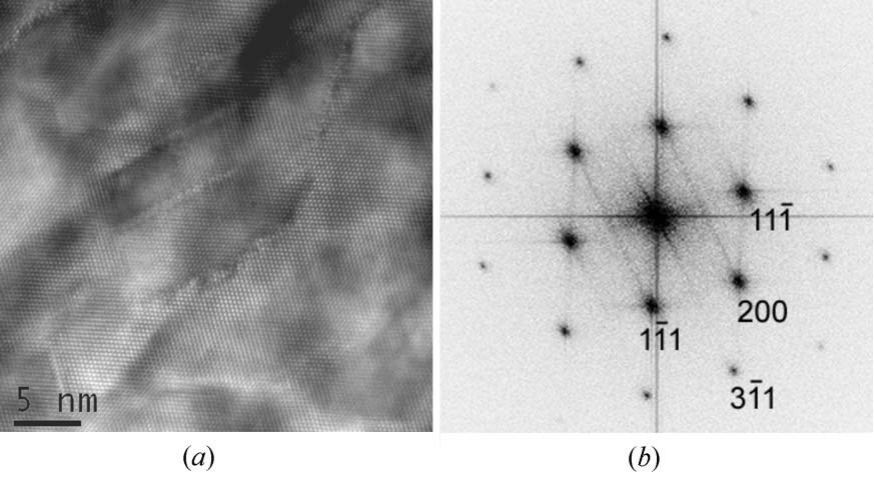
{113} defects in an Si thin film. (*a*) HRTEM image in cross section in the [011] zone axis and (*b*) power spectrum. The defects are situated in the (3
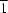
1) plane as shown by the streaks in the power spectrum.

The Si thin films deposited onto a glass substrate covered by an SiN layer are polycrystalline and contain many planar defects. All the grains also exhibit odd EDPs. Details are given in §4.3. HRTEM images, such as the one presented in Fig. 9 ▶, are very informative on the nature of the defects. In the very thin parts of the sample, alternating twins on the {111} planes are clearly visible, but in the thicker parts the twins cannot be observed anymore and are replaced by patterns that look like the superstructure with the periodicity of 3*d*_(111)_ already observed in the Si NW. However, the ‘superstructure-like’ patterns in the Si thin films (Fig. 9 ▶) are not as clear and periodic as those obtained in the Si NWs (Fig. 7 ▶) and, actually, they look more like Moiré patterns than real superstructure. The interpretation is in §5. Other planar defects on the {113} planes could also be identified by HRTEM in the thick parts of the sample (Fig. 10 ▶). Such defects have already been identified and studied by Parisini & Bourret (1993 ▶).

### 4.3. Odd EDPs in Si NWs and Si thin films

**Figure 11:**
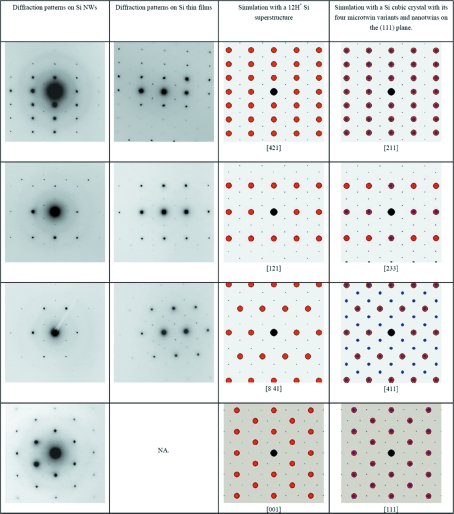
Odd EDPs acquired on Si NWs (first column) and on Si thin films (second column), and their simulations with a hypothetical 12H* superstructure (third column) and with a normal cubic Si structure with diffraction effects produced by microtwins and nanotwins (fourth column). In these simulations, we have considered that the four microtwin variants diffract (blue discs). The extra spots due to the streaking effects produced by the nanotwins have been calculated with a unique degeneracy vector **s** = 3**g**_(111)_ (black dots). The four simulated zone axes are [211], [233], [411] and [111] (in the 3C cubic basis). The zone axes presented in this figure do not allow unambiguous discrimination between the two hypotheses (hexagonal superstructure or twin diffraction artifacts).

**Figure 12:**
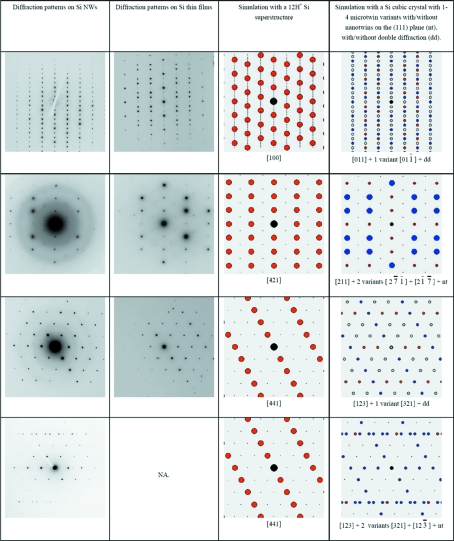
Odd EDPs acquired on Si NWs (first column) and on Si thin films (second column), and their simulations with a hypothetical 12H* superstructure (third column) and with a normal cubic Si structure with diffraction effects produced by microtwins and nanotwins (fourth column). The microtwin diffraction spots are represented by blue discs and the nanotwin spots created by streaking are represented by black dots. The spots generated by double diffraction between the microtwins and the parent crystal are represented by grey discs. The simulated zone axes are [011], [211] and [123] (in the 3C cubic basis). The EDPs of lines 3 and 4 are very similar – both are EDPs on the [123] zone axis. The only difference is the presence of doublets in the EDPs of line 4 due to the presence of at least two twin variants. The intensities of the diffraction spots in this figure allow us to conclude unambiguously that the odd EDPs are due to the microtwins and nanotwins, and not to a hexagonal superstructure of silicon.

The odd experimental EDPs are presented in Figs. 11 ▶ and 12 ▶, in the first column for the Si NWs and in the second column for the Si thin films. It is possible to see the great similarities between the odd EDPs in these two materials. Some of these patterns clearly correspond to the normal cubic Si structure, but with additional spots (Fig. 11 ▶, column 1 or 2). Other EDPs cannot be easily indexed with the diamond Si structure (Fig. 12 ▶, column 1 or 2) because some interplanar distances (such as 0.33 nm) and angles (such as 61°) do not match those of Si. The extra spots at 1/3{224} that have been reported by other authors (see end of §1) are visible in Fig. 11 ▶, last line. As discussed in §1, different explanations are possible. The fact that the odd EDPs are observed even for NWs larger than 100 nm and the similarities between those obtained on the Si NWs and on the Si thin films allow us to conclude that these EDPs are not due to size effects as suggested by some authors (§1.1). This leaves the possibilities that they are due to diffraction artifacts produced by twins (see §1.2) or to the existence of a new structure of silicon (§1.3).

## 5. Simulations and discussion

### 5.1. Odd EDPs are not due to a silicon superstructure

At the start of this study, we believed a hexagonal superstructure existed in the Si NWs and we tried to determine it according to the EDPs of Figs. 11 ▶ and 12 ▶ (first column). We quickly realized that the wurtzite structure (2H) proposed by some authors (Zhang *et al.*, 1999 ▶; Fontcuberta i Morral *et al.*, 2007 ▶) was not appropriate because of the very large discrepancy in the positions of the diffraction spots between the experimental and theoretical patterns (> 5%) and the impossibility of reproducing the intensities in these patterns. The EDP and the power spectrum obtained along the 〈110〉 zone axis (Fig. 12 ▶, first column, first line, and Fig. 7 ▶ *b*) led us to attempt hexagonal structures with a ratio *c*/*a* = 3σ, *i.e.* some phases derived from the normal 3C cubic diamond phase, but without success. Better agreement could be obtained with the 6H-Si polytype structure (with the sequence *ABCBAC*) proposed by Miyamoto & Hirata (1978 ▶), but some extra spots in the EDPs of Fig. 11 ▶ (first column) were completely missing in the simulations. We also tried to simulate the EDPs with a 9R-Si structure because of the similarities of the HRTEM images of Fig. 7 ▶ (or Figs. 8 ▶ and 9 ▶) with published images of 9R-GaN polytypes (Selke *et al.*, 2000 ▶), still without success. Finally, we discovered that the most appropriate structure that reproduces all the positions of the diffraction spots of Figs. 11 ▶ and 12 ▶ with accuracy greater than 1% is a hexagonal structure with lattice parameters *a*_H_ = 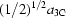 and *c*_H_ = 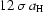. However, the 12H-Si polytype (with stacking sequence *ABCABCBACBAC*) does not explain the intensities of the experimental EDPs. Thus, we imagined different superstructures with *c*/*a* = 12σ:

(i) By adding Si in interstitial positions in the tetrahedral and hexagonal sites (Baryam & Joannopoulos, 1984 ▶). Supersaturated Si is possible as a result of the high cooling rate of Si at the Au–Si eutectic point.

(ii) By creating Si—H bonds. Retained Si—H bonds in the Si NW are conceivable if the silane gas is not completely dissociated during the VLS mechanism. Moreover, hydrogen-induced platelets have already been observed by HRTEM in Si on the {111} planes by Muto *et al.* (1995 ▶). Many different types of Si—H structures have been reported in the literature (Mainwood & Stoneham, 1984 ▶; Zhang & Jackson, 1991 ▶).

More than 30 superstructures representing all the possibilities offered by these hypotheses were simulated and compared with the experimental EDPs. Despite our efforts, no structure could match the intensities. Moreover, additional Raman measurements were later performed on our Si NWs, and neither the Si—H nor the H_2_ peaks could be detected at 2100 or 3600 cm^−1^, respectively (Leitch *et al.*, 1998 ▶; Ishioka *et al.*, 2003 ▶). Finally, we found that the ‘best’ superstructure with the ratio of lattice parameters *c*/*a* = 12σ is, in fact, a phase very close to the normal cubic Si (3C) with only an infinitesimal shift of the atomic positions each 12(111) planes, *i.e.* a structure with sequence A*BCABCABCABC (the symbol * meaning here ‘slightly shifted’) that will be denoted 12H*. The zone axes of the 12H* structure were determined by (i) ignoring the extra spots in the EDPs of Fig. 11 ▶, (ii) indexing the EDPs with the 3C structure, (iii) determining the zone axis in the 3C basis, and finally (iv) transforming this zone axis in the reference frame of the 12H* phase according to the matrix of equation (1) of §3.1. For example, we found that [211]_3C_ = [421]_12H*_ and [233]_3C_ = [121]_12H*_. We have simulated the experimental EDPs of Figs. 11 ▶ and 12 ▶ with the 12H* structure. A very good agreement is obtained between the experimental EDPs of Fig. 11 ▶ and the simulations (compare the first or second column with the third column). All the positions of the diffraction spots in Figs. 11 ▶ and 12 ▶ are simulated with an accuracy greater than 1%. However, we were still unable to explain the intensities of some EDPs, as illustrated in Fig. 12 ▶ (compare the first or second column with the third column). We have to conclude that neither a polytype nor any other hexagonal superstructure of silicon could satisfactorily explain the EDPs obtained in the Si thin films and Si NWs investigated in our studies. It was mentioned in §1.3 that silicon polytypes may exist in the Si thin films (and possibly in the Si NWs), but they correspond only to local structures limited to a few atomic planes at twin intersections (Dahmen *et al.*, 1989 ▶) or to short sequences of stacking faults (Cerva, 1991 ▶). We have seen in this section that neither these polytypes nor other superstructures can explain the odd EDPs. There are no massive 2H-Si or other Si polytypes in the Si thin films and Si NWs presented in this study.

### 5.2. Odd EDPs are explained by micro- and nanotwinning

Now, let us consider the last hypothesis (§1.3). We have simulated with *GenOVa* the experimental EDPs by assuming that the grains in the Si thin films and in the nanowires are constituted of crystals with an fcc Si structure that contains (i) microtwins and (ii) nanotwins on the {111} planes. We have also assumed that double diffraction is possible. Both positions and intensities are perfectly reproduced, as can be seen in Figs. 11 ▶ and 12 ▶ by comparing the experimental EDPs (first and second column) with the simulations (fourth column). In Fig. 11 ▶, the simulations were performed by assuming the existence of the four twinning variants and nanotwins on only one of the four {111} planes, without double diffraction. The zone axes of the twins were calculated according to the matrices of equation (4) of §3.3.

In Fig. 11 ▶, the spots created by the four microtwin variants superimpose exactly with the matrix spots. All the extra spots on this figure come from the nanotwins (or stacking faults) and not from the microtwins. Let us consider two examples. In the first example (Fig. 11 ▶, last line), the extra spots 1/3{422} visible in the [111] zone axis come from the degeneration of the HOLZ spots along the reciprocal direction **g**_(111)_ by the equality 1/3(22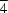) = (11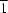) − 1/3(111), with (11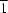) ∈ FOLZ (first-order Laue zone). This explanation confirms the hypothesis of Kohno *et al.* (2003 ▶). We believe that the extra spots at 1/3{422} observed by Carim *et al.* (2001 ▶) in the power spectrum of an Si NW oriented along a [111] zone axis come from this streaking effect (probably due to {111} planar defects) and not, as assumed by these authors, from the microtwins sometimes observed along the nanowires. We think that the extra spots at 1/3{422} that appear in their simulations are due, in fact, to an excessively low diameter chosen for the nanowire which produces a size effect by incomplete sequence packing (see §1.1). Such size effects are not relevant in their (and our) Si NWs because the diameters of the Si NWs observed experimentally are larger than 80 nm. The streaking effect is simpler than the notion of double diffraction without excited spots suggested by Pashley & Stowell (1963 ▶), and we think that the 1/3{422} extra spots observed by these authors at grain boundaries in gold thin films come, in fact, from a similar streaking effect produced by nano-stepped interfaces between the interpenetrated grains as represented in Fig. 1 ▶(*b*). In the second example (Fig. 11 ▶, first line), the extra spots visible in the [211] zone axis come from the degeneration of the HOLZ spots along the reciprocal direction **g**_(111)_ by the equality 1/2(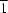
3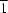) = (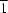
1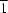) + 1/2(111), with (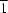
1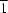) ∈ SOLZ (second-order Laue zone), and not from diffraction of the twin variants as assumed by Kohno *et al.* (2003 ▶).

Other EDPs cannot be explained by the streaking effect. In these cases, the extra spots are due to the microtwins and double diffraction. In Fig. 12 ▶, the simulations have been performed by adjusting the number of variants (between one and four) and by allowing double diffraction. Double diffraction between a pair of twinned crystals explains the [123] zone axis EDP presented in Fig. 12 ▶, third line, as already proved by Dickson *et al.* (1964 ▶) and later by Kohno *et al.* (2003 ▶). Sometimes, more than two twin variants can diffract to form more complex EDPs, as shown in Fig. 12 ▶, fourth line (please note the presence of double spots in contrast to the same zone axis presented in the third line). Our simulations also prove that the extra spots at 1/3(111) in the [110] zone axis EDP (Fig. 12 ▶, last line) come from a double diffraction between the Si crystal and one of its microtwins. This double diffraction effect explains the superstructure-like Moiré patterns of Figs. 7 ▶, 8 ▶ and 9 ▶. The Σ3 misorientation between the parent crystal and its microtwin is so exactly respected in the Si NWs that the superposition of the twinned crystals mimics a pattern that looks like a superstructure. Following this idea, we could simulate the superstructure-like pattern of Fig. 8 ▶ simply by superposing, with the help of Photoshop, the two parts of the twinned crystal of Fig. 4 ▶(*b*). The ‘simulation’ is represented in the square at the top right of Fig. 8 ▶. The Σ3 misorientation is not so accurately respected in Si thin films (probably because of the high stress level in these films), and the HRTEM images are less misleading and can be directly interpreted as Moiré patterns (Haji *et al.*, 1994 ▶). Other complex EDPs can also be observed and simulated as a superposition of an Si matrix with two twin variants, or by twins of first and second orders, *i.e.* by crystals linked by Σ3 and Σ9 operators (figures not shown).

### 5.3. Stopping the microtwins

**Figure 13:**
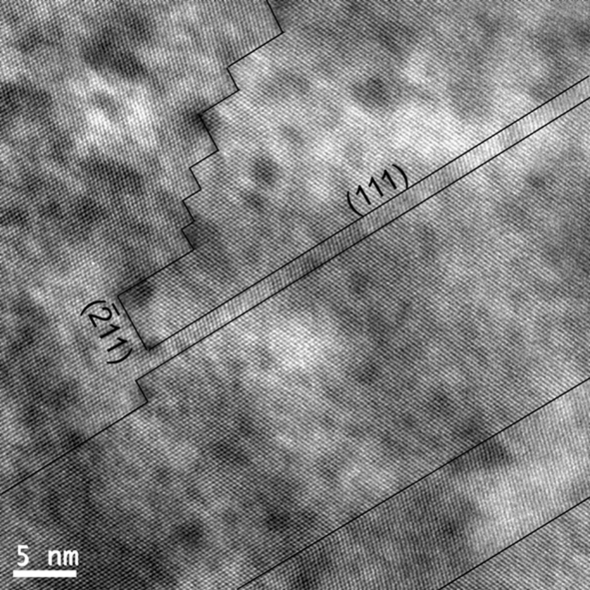
HRTEM image of a trench of a twinned Si NW prepared by FIB cross section. The zone axis is [01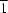]; it is also the NW growth direction. The main interface plane between the matrix and its twin is the common (111) plane. A twin stops freely on a surface constituted of edges on the common (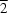
11) planes.

**Figure 14:**
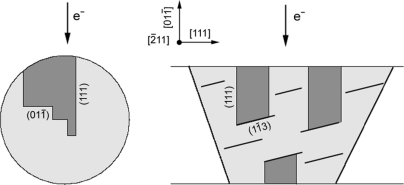
Cross-section schemes of overlapping twins in Si NWs and in Si thin films. In the Si NWs, the twins can freely terminate in the matrix or can be stopped by other microtwins or by nanotwins. In Si thin films, we think that the twins are stopped by other microtwins or by {113} planar defects. The overlapping is at the origin of the superstructure-like patterns of Figs. 7 ▶, 8 ▶ and 9 ▶.

Double diffraction and the associated Moiré effect observed in the 〈110〉 zone axis are possible only if the twinned crystals are superposed along their common 〈110〉 direction. Such overlapping is unusual because, most often, the interface plane is the (111) mirror plane and the common 〈110〉 directions belong to this plane, as is the case in Fig. 4 ▶(*a*). Therefore, the interface plane between the twinned crystals observed in the Si NWs (Figs. 7 ▶ and 8 ▶) and Si thin films (Fig. 9 ▶) is not the usual (111) mirror plane. This then begs the question, which plane is it? In order to clarify this point, an Si NW was prepared by FIB cross section. In Fig. 13 ▶, twins are clearly visible and one of them stops inside the matrix without peculiar microstructural obstacle. The stopping interface is constituted of edges on the common {112} plane. However, one cannot generalize this example. For example, in the Si NW presented in Fig. 7 ▶, the contrast is continuous along the common 〈112〉 growth direction, which implies that the interface plane contains this direction: it can be {110} or {113} but not {112}. The microtwins can also be blocked by the other microtwins or nanotwins. In the Si thin films, it is probable that the numerous {113} planar defects (Fig. 10 ▶) act as barriers for the microtwins. A schematic representation is given in Fig. 14 ▶. Further work is required to determine statistically the interface planes of the overlapping twins in the Si NWs and Si thin films.

## 6. Conclusion and perspectives

Some silicon nanowires grown *via* the VLS method and Si thin films deposited by electron beam evaporation show odd transmission electron diffraction patterns. Three distinct explanations coexist in the literature and we decided to clarify this issue. To this end, the microstructures of Si NWs and Si thin films have been characterized by TEM, HRTEM and HRSTEM. The Si NWs have different shapes: they can be straight and monocrystalline with 〈111〉 or 〈110〉 growth directions, bent and twinned along their 〈112〉 growth direction, or multi-branched and multiply twinned. The Si NWs contain microtwins and also nanotwins, *i.e.* very thin twins containing less than ten (111) planes. The polycrystalline Si films contain many {113} planar defects. Both Si NWs and Si thin films show comparable odd EDPs and superstructure-like patterns in the HRTEM images. The similarities between these two different silicon materials prove that the odd EDPs are not artifacts resulting from the size of the objects, as assumed by some authors. 

We have simulated the EDPs according to the two remaining hypotheses, *i.e.* existence of a hexagonal phase or twinning effects. We found that most of the EDPs can be simulated with a hexagonal structure with a ratio of lattice parameters *c*/*a* = 12σ; however, we were still unable to simulate the intensities of the diffraction spots. Finally, we established that the odd EDPs are, in fact, explained by a normal cubic silicon structure with additional twinning effects. There are two kinds of extra spots. The first type are created by double diffraction between the Σ3 microtwins superposed along the electron beam direction. Such overlapped twins are not easy to identify because the interface plane is not the usual (111) mirror symmetry plane. The superstructure-like patterns that are sometimes visible on the HRTEM and HRSTEM images are, in fact, Moiré patterns resulting from this superposition. The other type of spots, such as the 1/3{422} spots, are created by a streaking effect produced by the nanotwins. We conclude that, despite what is widely accepted in the scientific community, there is no bulk hexagonal phase in the Si NWs and Si thin films. Such exotic phases reported in the literature probably exist but are limited to an ordering of a few atomic (111) planes and do not explain the odd EDPs.

This result improves our understanding of, and hence our ability to optimize, the physical properties of the Si NWs and Si thin films that will be integrated in the future solar cells and thermoelectric devices. Micro- and nanotwins do not necessarily have to be considered as defects; for example, Lu *et al.* (2004 ▶) proved that it is possible to improve the mechanical and physical properties of copper thin films by increasing the twin densities. Moreover, Σ3{111} twins could probably be used as electron/phonon filters in thermoelectric devices thanks to their very low electrical resistivity in comparison with a random grain boundary (Sutton & Balluffi, 1995 ▶) and their probable influence as phonon barriers, although, to our knowledge, there are no experimental data on this point. Further experiments and simulations on phonon transport by twins are required before considering Σ3 grain boundary engineering as a new way of improving the thermoelectric figure of merit of silicon.
